# Increased prevalence of hypertension in Ghana: New 2017 American College of Cardiology/American Hypertension Association hypertension guidelines application

**DOI:** 10.7189/jogh.10.020408

**Published:** 2020-12

**Authors:** Sampson Opoku, Emmanuel Addo-Yobo, Diana Trofimovitch, Rebekah Bless Opoku, Joseph Lasong, Yong Gan, Zuxun Lu

**Affiliations:** 1 Department of Social Medicine and Health Management, School of Public Health, Tongji Medical College, Huazhong University of Science and Technology, Wuhan, China; 2 Department of Medicine, SUNY Upstate Medical University, New York, USA; 3 Department of Internal Medicine, East Tennessee State University, Johnson City, Tennessee, USA; 4 Community 8, Number 3, Junior High School, Ghana Education Service, Tema, Ghana; 5 Institute of Reproductive Health, Tongji Medical College, Huazhong University of Science and Technology, Wuhan, China

## Abstract

**Background:**

We estimated the prevalence and socio-demographic risk factors of hypertension among Ghanaian adults as per the Joint National Committee 7 and the 2017 American College of Cardiology/American Hypertension Association hypertension thresholds used for diagnosis and treatment.

**Methods:**

This cross-sectional analysis included 12 151 adults (8295 females and 3856 males) aged 18 years or older who participated in the 2014 Ghana Demographic and health Survey. Multiple logistic regression models were applied to obtain risk factors associated with hypertension as per both guidelines.

**Results:**

Overall, 30.43% (n = 3698) and 11.48% (n = 1395) respondents had hypertension as per the 2017 ACC/AHA and JNC7 guidelines, respectively. The following factors were significant according to the 2017 ACC/AHA guideline: 55-64 years (adjusted odds ratio (aOR) = 6.42, 95% confidence interval (CI): 4.70-8.77), 45-54 years (aOR = 5.72, 95% CI = 4.70-6.85), 3544 years (aOR = 3.91, 95% CI = 3.33-4.59), and 25-34 years (aOR = 2.05, 95% CI = 1.77-2.37) age groups. Males (aOR = 1.39, 95% CI = 1.23-1.53), and urban residents (aOR = 1.18, 95% CI = 1.05-1.38). All the above risk factors were significant according to the JNC7 guideline too. Factors positively associated with only the 2017 ACC/AHA guideline included: middle income (aOR = 1.20, 95% CI = 1.02-1.42) and richest (aOR = 1.36, 95% CI = 1.10-1.69) wealth quintiles, whereas manual (aOR = 1.37, 95% CI = 1.02-1.86) was positively associated with the JNC7 guidelines only.

**Conclusions:**

We conclude that adopting the ACC/AHA guidelines would lead to a substantial increase in the prevalence of hypertension among Ghanaian adults, thus, hypertension prevention and control should be prioritized.

Cardiovascular disease (CVD) is a major cause of morbidity and mortality with a high incidence and prevalence in countries of all economic groups [1]. One of the major risk factors of CVD is hypertension or high blood pressure (BP), and ranked first of the three leading risk factors for global disease burden in 2010 [2]. In addition, BP affects one in four persons globally, rendering it as the single most important risk factor for mortality and the third highest cause of morbidity [3]. Hypertension was previously regarded as a disease of low prevalence in the developing world, however, recent studies have shown that it is a huge burden for developing countries with increased risk of adverse outcomes [4,5]. A pooled analysis of global trends in blood pressure from 1975 to 2015 reveals that the highest levels of BP have shifted from high-income countries to low-income countries in South Asia and Sub-Saharan Africa (SSA) [6].

Previous studies on hypertension in Ghana have reported increases in prevalence and its significant effects on cerebro-vascular accidents (CVA), as well as coronary heart disease’s (CHD) morbidity and mortality in recent years [7,8]. These increased prevalence might be attributable to multiplicity of factors such as staying in urban areas and ageing [9,10].

In 2017, the American College of Cardiology/American Heart Association (ACC/AHA) came out with the current Guidelines for the Prevention, Detection, Evaluation, and Management of High BP in Adults. This latest guideline decreased the cutoffs for the definition BP. It describes the condition as a systolic blood pressure (SBP) greater than or equal to 130 mm Hg or a diastolic blood pressure (DBP) greater than or equal to 80 mm Hg, this is 10 mm Hg lower than the previous cut-off points. This lower threshold was categorized as the pre-hypertensive range per the previous guidelines; the recent guideline replaced the term “prehypertension” with “elevated blood pressure”[11].

Earlier guidelines, such as the WHO – International Society of Hypertension Guideline (1999 WHO-ISH) [12], and the Seventh Report of the Joint National Committee on Prevention, Detection, Evaluation, and Treatment of High Blood Pressure (JNC7), defined hypertension as SBP greater than or equal to 140 mm Hg or a DBP greater than or equal to 90 mm Hg [13].

Considering the latest BP definition, a sizeable number of adults would be re-categorized as hypertensive, who were previously classified as pre-hypertensive, or individuals with high normal blood pressure. A study by Muntner et al. in US has indicated that the new prevalence of hypertension among US population would be 14.7% higher compared with the previously recommended Joint National Committee 7 (JNC7) cut-off points of 140/90 mm Hg [14]. Again, the new ACC/AHA guideline has led scientist in other parts of the world to re-estimate and update the prevalence of hypertension and related risks factors to give more meaning and significance to the revised guideline [15,16]. For example, two studies in Bangladesh and Nepal investigated differences in hypertension prevalence by comparing the 2017 ACC/AHA and JNC7 guidelines, and reported that about 22.3% of individuals in Bangladesh, similar to Nepal’s findings would now be classified as having hypertension due to the lowering of the blood pressure cut-offs [17]. These observed increases in hypertensive cases as a result of the new guideline add to the reasons why more and more countries or regions in various parts of the world need to revise their estimates for prudent planning and implementation of BP management strategies.

Prevalence assists in disease burden estimation, whereas risk factors are helpful in identifying subpopulations who may benefit from specific health interventions [17]. Previous studies that measured BP with the earlier definition of 140/90 mm Hg found that hypertension prevalence could vary according to age, sex, level of education and other sociodemographic characteristics [11,15].

Considering the huge number of pre-hypertensive individuals who would now be referred to as hypertensives [11,17], the risk factors of this “new group” of hypertensives could also change, as previous studies found that “prehypertension” (as per previous guidelines) might vary according to background features of study respondents [18,19]. Modifying blood pressure level to define hypertension could also change the associated factors that affect prevalence or likelihood of hypertension. Earlier studies conducted in Ghana on BP prevalence and risk factors used WHO guidelines [12].

The JNC7 and ACC/AHA 2017 guidelines have not been used to estimate hypertension prevalence among Ghanaian adults with the GDHS 2014 data. The use of these guidelines to estimate and compare the prevalence and risk factors of hypertension for Ghana would be of particular interest and significance because not only it is the first for the country, but could also help to identify patients sooner, and probably lead to better management of hypertension in adults to minimize morbidities and mortalities in the country. Hence, the current study was conducted to estimate the associated socio-demographic risk factors and prevalence of hypertension among Ghanaian adults ≥18 years using the JNC7 and the 2017 ACC/AHA hypertension guidelines, and to compare these risk factors across the two guidelines using the GDHS data.

## METHODS

### Study setting

Ghana is a developing country centrally located on the West coast of Africa. It has a total land area of 238 537 km^2^. Ghana is a lowland country except for a range of highlands on the eastern border. Ghana can be divided into three ecological zones: the low, sandy coastal plains, the middle and western parts, and a northern savannah.

There are 10 administrative regions in Ghana: Western, Central, Greater Accra, Volta, Eastern, Ashanti, Brong Ahafo, Northern, Upper East, and Upper West. Ghana’s population was estimated at 27 million in 2014. The average annual growth rate between 2000 and 2010 was 2.5 percent.

The regions are subdivided into 216 districts to ensure equitable resource allocation and efficient, effective administration at the local level. The Ghanaian population is made up of several ethnic groups, with the Akans constituting the largest group (48 percent), followed by the Mole-Dagbani (17 percent), Ewe (14 percent), Ga-Dangme (7 percent), and others.

The 2014 GDHS is the sixth in a series of Demographic and Health Surveys conducted in Ghana. The main objective of the survey was to provide up-to-date information on various health indicators such as fertility, childhood mortality levels and prevalence of diseases among adult populations to assist in health care planning, policy making and to evaluate health interventions and strategies in Ghana [20].

### Data source

Secondary analysis was performed using data from the 2014 GDHS, which is the latest and largest nationally representative health survey publicly available in Ghana. It was implemented by the Ghana Statistical Service (GSS), the Ghana Health Service (GHS), and the National Public Health Reference Laboratory (NPHRL) of the GHS [21]. These data have been weighted to cater for the different sample proportions. The survey was conducted from early September to middle December, 2014 and included these structured questionnaires: the Women’s Questionnaire, the Household Questionnaire, and the Men’s Questionnaire.

### GDHS sample design and procedure

The details of the sampling procedure used in the GDHS have been previously published [19]. In short, the GDHS was a nationally representative, cross-sectional household survey, which adopted a stratified, two-staged probability sample design [19,20]. First, clusters (made up of enumeration areas (EAs)) of the 2010 Ghana Population and Housing Census (PHC) were delineated. This led to a total of 427 clusters throughout the country (216 clusters in urban areas and 211 clusters in rural areas). The second stage of the design involved household listing in all the selected EAs. A systematic sampling method was used to select about 30 households from each cluster resulting in the selection of 12 831 households countrywide. All men aged 15-59 and women aged 15-49 years who were either permanent residents of selected households or visitors who stayed in the households the night before the survey were eligible to be interviewed. In addition, blood pressure measurements were done for all the selected households for the female survey, but for the male survey, it was taken for half of the households selected [20]. A 99% response rate of all selected households was achieved. Considering eligible participants to be interviewed, 97% and 95% of women and men were interviewed, respectively [19].

### Analytic sample and population

The GDHS sample design uses different parameters for indicators to estimate the final sample size. In view of the above, we used recoded files of men and women (4388 and 9396), respectively. Specifically, our study focused on adults (men and women) 18 years and above.

### Study variables

#### Outcome variable

Hypertension was defined according to the two guidelines used in this study. First, the JNC 7 guideline defines the condition as individuals who have an SBP greater than or equal to 140 mm Hg or a DBP greater than or equal to 90 mm Hg or take any prescribed drugs to control blood pressure. Second, according to the 2017 ACC/AHA guideline, individuals who have an SBP greater than or equal to 130 mm Hg or a DBP greater than or equal to 80 mm Hg or take any prescribed drugs to control blood pressure. The category of prehypertension was changed to elevated blood pressure in the 2017 ACC/AHA guidelines (Table S1 in the **Online Supplementary Document**).

#### Blood pressure measurement

The blood pressure of the survey participants was measured with the LIFE SOURCE UA-767 Plus blood pressure monitor. It is a standardized device recommended by the WHO. Subjects sat quietly in a chair during blood pressure measurement. Three consecutive measurements were taken for each individual in a sitting position with an interval of 10 minutes or more between measurements. The measurements were taken by trained field workers. The exclusion criteria included: nonresidents ie, those who did not spent a night in a household during the census; individuals <18 years of age; residents who were absent during the survey; and those whose BP was not measured thrice. All values of BP measurements were checked and randomly cross-verified for consistency. In order to minimize bias that might be introduced as a result of making decision on blood pressure based on only one measurement, the average of the three recorded measurements of BP were calculated. Then the final blood pressure level was binary coded based on both criteria (JNC 7 and 2017 ACC/AHA) to define hypertension.

#### Independent variables

We obtained information on the following socio-demographic variables guided by studies in Ghana and other developing countries to determine the risk factors of hypertension [17,19-21].

Variables used are as follows: Individual level variables: sex (male, female); age (18-24 years, 25-34 years, 35-44 years, 45-54 years, 55 and above); marital status (never married, married, living with partner and Widowed/Divorced/No Longer Living Together & Separated (WD/DV/S); place of residence (rural, urban). Household level variables: region of residence (Western, Central, Greater Accra, Volta, Eastern, Ashanti, Brong Ahafo, Northern, Upper East, Upper West) and ethnicity (Akan, Ga-Adangbe, Ewe, Mole-Dagbani and other ethnic groups). The proxy indicators of socio-economic status: level of education (no education, primary, secondary, college or above); occupation (not working, professional/technical/clerical, sales, agriculture, and Household and Domestic/Services/Skilled Manual/ Unskilled manual, (HH/DD/S) and Wealth quintiles (poorest, poorer, middle, richer, richest). The wealth index was designed from household assets data using principal components analysis. These assets consisted of a television, bicycle, or car, as well as dwelling characteristics such as a source of drinking water, sanitation facilities, and type of flooring material. The other ethnic group’s category of the ethnicity variable was a pool of more than five smaller groups (Guan, Grusi, Gruma, Mande and others) in Ghana. Table S1 in the **Online Supplementary Document** describes all study variables and their categories.

### Statistical analysis

The demographic characteristics of the study participants were reported according to the presence of hypertension (as per both JNC7 and 2017 ACC/AHA guidelines), as well as the overall population. Since all independent variables were categorical, numbers and frequencies were used to summarize the data. The data set was considered to report weighted frequencies because of the two stage stratified cluster sampling design in DHS surveys. Identification of the significant associates of hypertension, as per both guidelines, was primarily done using bivariate logistic regression. All explanatory variables were considered to be nested in a cluster for multivariable analysis. Then, the significant variables (*P* < 0.05) were maintained for the multivariable logistic regression. Crude odds ratio and adjusted odds ratio were reported in the results. McNemmar’s test was used to check up the difference in labelling a patient as having hypertension using the two guidelines. Multicollinearity was assessed using variance inflation factor. *P*-values less than 0.05 were considered as significant throughout the analysis. Stata 13.0 (StataCorp, College Station, TX, USA) was used for data analysis. Processing of figures and tables was done using MS Excel 2013 (Microsoft Inc, Seattle, WA, USA).

## RESULTS

### P**opulation description**

**Table 1** describes the background characteristics of the study population according to the overall sample and the number of subjects with or without hypertension (as per both JNC7 and 2017 ACC/AHA guidelines). Variables were presented in numbers and percentages. A total of 12 151 respondents were included in the analysis. Majority of the participants 3981 (32.8%) were found between the ages of 25 and 34 years, while adults aged 35-44 years recorded the highest number of hypertension cases; 529 (37.9%) for the JNC7, and 1275 (34.5%) for the 2017 ACC/AHA guidelines. More than half of the subjects were females (69.6%), and had a higher proportion of hypertension. Most respondents not only attained secondary level education 6358 (52.3%), but also had the highest number of BP cases with respect to both guidelines. Expectedly, hypertension was hugely prevalent among richest participants as per both guidelines with regards to the wealth quintile, JNC7 = 350 (25.1%) and ACC/AHA 2017 = 850 (23.0%).

**Table 1:** Participants characteristics n (%)

Characteristics	Overall No (12 151)		JNC7 No = (1395)	2017/ACC/AHA No = (3698)
**Age (in years):**				
18-24	3203 (26.4)		88 (6.3)	481 (13.0)
25-34	3981 (32.8)		316 (22.7)	1066 (28.8)
35-44	3206 (26.4)		529 (37.9)	1275 (34.5)
45-54	1530 (12.6)		389 (27.9)	751 (20.3)
55 +	231 (1.9)		73 (5.2)	125 (3.4)
**Sex:**				
Female	8295 (68.3)		811 (58.1)	2323 (62.8)
Male	3856 (31.7)		584 (41.9)	1375 (37.2)
**Education:**				
No formal education	2872 (23.6)		297 (21.3)	799 (21.6)
Primary	1963 (16.2)		208 (14.9)	566 (15.3)
Secondary	6358 (52.3)		741 (53.1)	1991 (53.8)
College+	958 (7.9)		149 (10.7)	342 (9.2)
**Wealth quintile:**				
Poorest	2956 (24.3)		211 (15.1)	657 (17.8)
Poorer	2260 (18.6)		216 (15.5)	626 (16.9)
Middle	2407 (19.8)		282 (20.2)	742 (20.1)
Richer	2335 (19.2)		336 (24.1)	823 (22.3)
Richest	2193 (18.0)		350 (25.1)	850 (23.0)
**Marital status:**				
Never married	3303 (27.2)		184 (13.2)	697 (18.8)
Married	6198 (51.0)		875 (62.7)	2108 (57.0)
Living with partner	1535 (12.6)		138 (9.9)	421 (11.4)
WD/DV/S	1115 (9.2)		198 (14.2)	472 (12.8)
**Place of residence:**				
Urban	5958 (49.0)		846 (60.6)	2093 (56.6)
Rural	6193 (51.0)		549 (39.4)	1605 (43.4)
**Occupation:**				
Not working	1913 (15.8)		107 (7.7)	394 (10.7)
Prof./Tech. M/Clerical	1072 (8.8)		187 (13.4)	403 (10.9)
Sales	3195 (26.4)		430 (30.9)	1123 (30.5)
Agriculture	3568 (29.4)		338 (24.3)	944 (25.6)
HH/DD/S	2373 (19.6)		331 (23.8)	824 (22.3)
**Region:**				
Western	1358 (11.2)		162 (11.6)	456 (12.3)
Central	1183 (9.7)		148 (10.6)	364 (9.8)
Greater Accra	1340 (11.0)		209 (15.0)	542 (14.7)
Volta	1026 (8.4)		139 (10.0)	329 (8.9)
Eastern	1207 (9.9)		133 (9.5)	354 (9.6)
Ashanti	1319 (10.9)		205 (14.7)	444 (12.0)
Brong Ahafo	1297 (10.7)		143 (10.3)	394 (10.7)
Northern	1344 (11.1)		98 (7.0)	302 (8.2)
Upper East	1152 (9.5)		92 (6.6)	268 (7.2)
Upper West	925 (7.6)		66 (4.7)	245 (6.6)
**Ethnicity:**				
Akan		4993 (41.1)	674 (48.3)	1676 (45.3)
Ewe		1472 (12.1)	201 (14.4)	497 (13.4)
Ga Adangbe		726 (6.0)	92 (6.60	256 (6.9)
Mole-Dagbani		2873 (23.6)	244 (17.5)	732 (19.8)
Others		2087 (17.2)	184 (13.2)	537 (14.5)

JNC *–* National Committee, ACC/AHA *–* American College of Cardiology/American Heart Association; WD/DV/S *–* Widowed/Divorced/No Longer Living Together & Separated; HH/DD/S *–* Household and Domestic/Services/Skilled Manual/Unskilled manual

With respect to marital status, married individuals constituted the highest proportion of respondents for both guidelines. If we turn to place of residence, 51% of the subjects stayed in rural areas, yet, urban dwellers recorded more cases of hypertension in relation to the guidelines. Considering both guidelines, Greater-Accra region had the highest proportion of adults with hypertension, (JNC7 = 15.0%; ACC/AHA 2017 = 14.7%). Most participants were Akans (41.1%), followed by Mole-Dagbanis (23.6%).

### Overall prevalence of hypertension per 2017 ACC/AHA and JNC7 guidelines

The prevalence of hypertension as per 2017 ACC/AHA and JNC7 guidelines were 30.43% and

**Figure 1:**
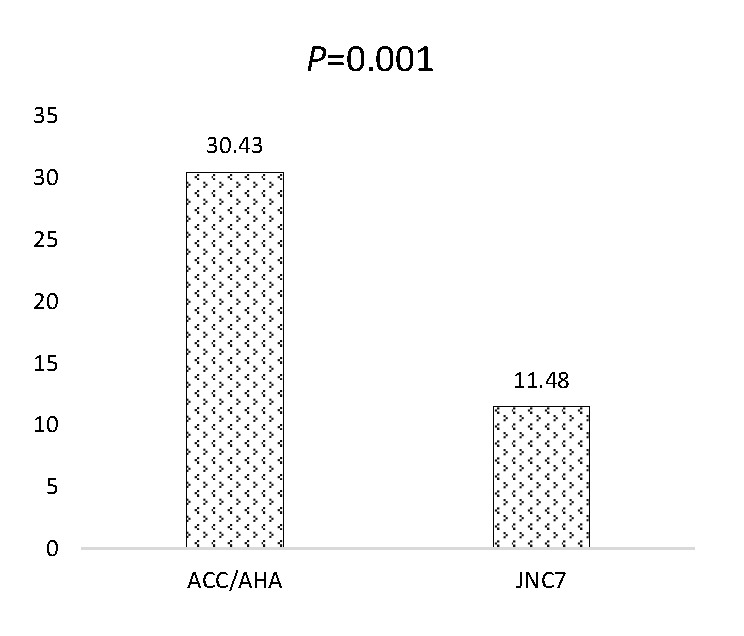
Prevalence of hypertension as per 2017ACC/AHA and JNC7 guidelines.

11.48%, respectively. Using the 2017 ACC/AHA guideline, about 2303 respondents were grouped as hypertensives, these people would have been classified as normotensives using the earlier guideline. Possible differences in grouping between the two guidelines was investigated using McNemmar’s test. The result showed that there is a significant difference in the final grouping of the respondents as hypertensive or not (*P* < 0.001) (**Figure 1****)**.

**Figure 2:**
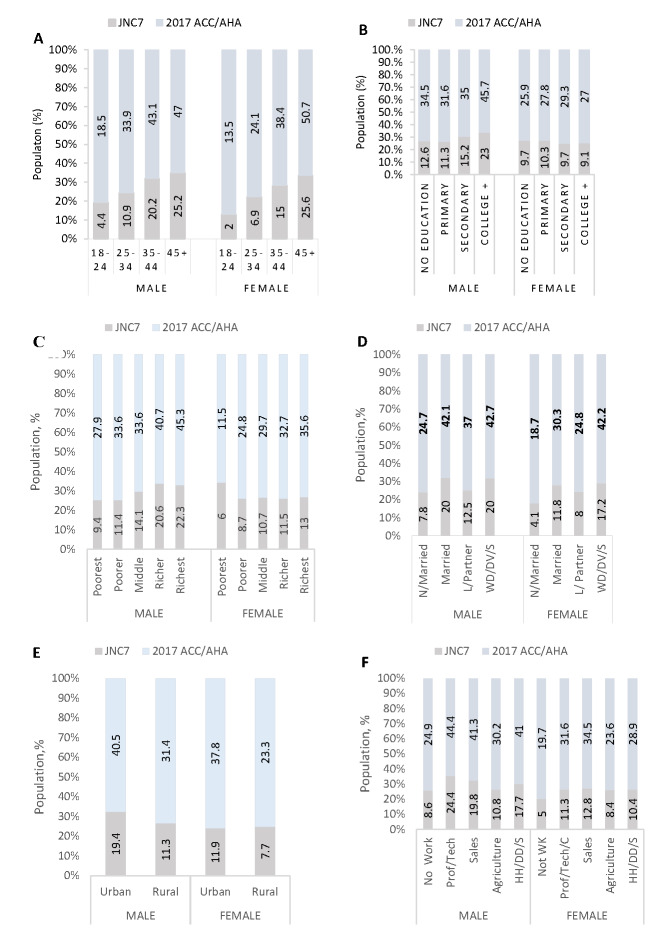
Prevalence of hypertension using the two guidelines stratified by sex. Panel A. Age groups. Panel B. Education level. Panel C. Wealth quintile. Panel D. Marital status (WD/DV/S = Widowed/Divorced/No Longer Living Together & Separated). Panel E. Place of residence. Panel F. Occupations. Panel G. Ethnic groups. Panel H. Administrative regions. Panel F. Occupation (HH/DD/S – Household/Domestic/Services/Skilled Manual/Unskilled manual). Panel H. Regions. 1 – Western, 2 – Central, 3 – Greater Accra, 4 – Volta, 5 – Eastern, 6 – Ashanti, 7 – Brong Ahafo, 8 – Northern, 9 – Upper East, 10 – Upper West.

**Figure 2** shows the prevalence of hypertension among males and females according to the two guidelines. Males compared with females had a significantly higher prevalence of hypertension as per the 2017 ACC/AHA guideline than the JNC7 guideline. Similar trends were observed in all the remaining risk factors per both guidelines stratified by sex.

### Risk factors of hypertension

In order to determine the risk factors of hypertension, both bivariate and multivariable regression analyses were done for the two guidelines. At bivariate level, all variables were found to be significantly associated with the presence of hypertension for both guidelines. Hence, they were incorporated in the multivariable analyses.

The multivariable logistic regression model using the JNC7 guideline showed that age was significantly associated with hypertension, with the highest OR of 13.91 (95% CI = 9.24-20.95) for 55-64 year-group, followed by an OR of 11.73 (95% CI = 8.68-15.84) for 45-54 year-group, 6.77 (95% CI = 5.08-9.02) for 35-44 year-group, and 2.82 (95% CI = 2.14-3.70) for 25-34 year-group. However, subjects’ level of education, marital status, region of residence, and the ethnicity of participants were insignificant per this guideline.

The odds of being hypertensive was 1.50 times among males compared to females. (aOR = 1.50; 95% CI = 1.30-1.73). Richer adults of the wealth index group were 1.40 times (aOR = 1.06-1.85) more likely to have hypertension as compared to poorest adults. Urban residents were found to have a significantly higher odds of BP (aOR = 1.27; 95% CI = 1.08-1.50) than rural residents.

Being a professional or a technical manager, or a clerical staff was a determinant of hypertension (aOR = 1.37; 95% CI = 1.02-1.86).

With regards to the 2017 ACC/AHA guideline, age was significantly associated with hypertension, even though the magnitude of associations of the age groups with hypertension were lesser compared to the JNC7 guideline. **(****Table 2****)**. Subjects in the following age groups: 25-34 (aOR = 2.05; 95% CI = 1.77-2.37), 35-44 (aOR = 3.91; 95% CI = 3.33-4.59), 45-54 (aOR = 5 72; 95% CI = 4.78-6.85), and 55-64 (aOR = 6.42; 95% CI = 4.70-8.77) were associated with BP. Males were more likely to have hypertension, 1.39 (95% CI = 1.26- 1.53). With regards to wealth quintile: middle, richer and richest adults were more likely to get BP (*P* < 0.05, *P* < 0.001). Participants residing in urban areas, and the Widowed/Divorced/No Longer Living Together & Separated had higher odds of getting hypertension, (aOR = 1.18; 95% CI 1.05-1.32) and (aOR = 1.27; 95% CI 1.06-1.52), respectively.

**Table 2:** Bivariate and multivariate logistic regression analyses of the risk factors associated with hypertension according to both guidelines

Characteristics	COR (95% CI)	2017 ACC/AHA	aOR (95% CI)	2017 ACC/AHA	
**Age (in years):**					
18-24	Ref.	Ref.	Ref.	Ref.	
25-34	3.05‡ (2.40, 3.88)	2.07‡ (1.84, 2.33)	2.82‡ (2.14, 3.70)	2.05‡ (1.77, 2.37)	
35-44	6.99‡ (5.55, 8.82)	3.74‡ (3.31, 4.21)	6.77‡ (5.08, 9.02)	3.91‡ (3.33, 4.59)	
45-54	12.07‡ (9.48, 15.36)	5.46‡ (4.75, 6.27)	11.73‡ (8.68, 15.84)	5.72‡ (4.78, 6.85)	
55+	16.36‡(11.54,23.19)	6.67‡ (5.06, 8.80)	13.91‡ (9.24, 20.95)	6.42‡ (4.70, 8.77)	
**Sex:**					
Female	Ref.	Ref.	Ref.	Ref.	
Male	1.65‡ (1.47, 1.85)	1.43‡ (1.31, 1.55)	1.50‡ (1.30, 1.73)	1.39‡ (1.26, 1.53)	
**Education:**					
No formal education	Ref.	Ref.	Ref.	Ref.	
Primary	1.03 (0.85, 1.24)	1.05 (0.93, 1.19)	1.01 (0.82, 1.24)	1.06 (0.92, 1.22)	
Secondary	1.14 (0.99, 1.32)	1.18† (1.07, 1.30)	0.98 (0.81, 1.18)	1.11 (0.97, 1.26)	
College +	1.60‡ (1.29, 1.97)	1.44‡ (1.23, 1.68)	0.94 (0.69, 1.27)	0.99 (0.79, 1.24)	
**Wealth quintile:**					
Poorest	Ref.	Ref.	Ref.	Ref.	
Poorer	1.38† (1.13, 1.68)	1.34‡ (1.18, 1.52)	1.04 (0.83, 1.31)	1.11 (0.96, 1.29)	
Middle	1.73‡ (1.43, 2.08)	1.56‡ (1.38, 1.76)	1.26 (0.99, 1.61)	1.20* (1.02, 1.42)	
Richer	2.19‡ (1.82, 2.62)	1.91‡ (1.69, 2.15)	1.40* (1.06, 1.85)	1.32† (1.09, 1.59)	
Richest	2.47‡ (2.06, 2.96)	2.22‡ (1.96, 2.50)	1.34 (0.98, 1.83)	1.36† (1.10, 1.69)	
**Marital status:**					
Never in union	Ref.	Ref.	Ref.	Ref.	
Married	2.79‡ (2.36, 3.29)	1.93‡ (1.75, 2.13)	1.07 (0.86, 1.33)	1.00 (0.87, 1.15)
Living with partner	1.67‡ (1.33, 2.11)	1.41‡ (1.23, 1.63)	0.91 (0.70, 1.18)	0.94 (0.80, 1.10)
WD/DD/S	3.66‡ (2.96, 4.53)	2.75‡ (2.37, 3.17)	1.22 (0.94, 1.58)	1.27† (1.06, 1.52)
**Place of residence:**				
Rural	Ref.	Ref.	Ref.	Ref.
Urban	1.70‡ (1.52, 1.91)	1.55‡ (1.43, 1.67)	1.27† (1.08, 1.50)	1.18† (1.05, 1.32)
**Occupation:**				
Not working	Ref.	Ref.	Ref.	Ref.
Prof/Tech M/Clerical	3.57‡ (2.78, 4.58)	2.32‡ (1.97, 2.74)	1.37* (1.02, 1.86)	1.15 (0.94, 1.40)
Sales	2.63‡ (2.11, 3.27)	2.09‡ (1.83, 2.39)	1.14 (0.90, 1.45)	1.15 (0.99, 1.34)
Agriculture	1.77‡ (1.41, 2.21)	1.39‡ (1.21, 1.59)	0.79 (0.60, 1.04)	0.81* (0.68, 0.96)
HH/DD/S	2.74‡ (2.18, 3.43)	2.05‡ (1.78, 2.36)	1.04 (0.81, 1.34)	1.03 (0.88, 1.20)
**Region:**				
Western	Ref.	Ref.	Ref.	Ref.
Central	1.06 (0.83, 1.34)	0.88 (0.74, 1.04)	0.97 (0.76, 1.25)	0.82* (0.69, 0.98)
Greater Accra	1.36† (1.09, 1.70)	1.34‡ (1.15, 1.57)	1.13 (0.88, 1.46)	1.11 (0.92, 1.33)
Volta	1.16 (0.91, 1.48)	0.93 (0.79, 1.11)	1.25 (0.91, 1.72)	0.96 (0.76, 1.20)
Eastern	0.91 (0.72, 1.17)	0.82* (0.69, 0.97)	0.92 (0.71, 1.19)	0.80* (0.66, 0.96)
Ashanti	1.36† (1.10, 1.70)	1.00 (0.86, 1.18)	1.21 (0.96, 1.53)	0.88 (0.74, 1.05)
Brong Ahafo	0.92 (0.72, 1.16)	0.86 (0.73, 1.02)	1.02 (0.79, 1.32)	0.95 (0.80, 1.14)
Northern	0.58‡ (0.45, 0.76)	0.57‡ (0.48, 0.68)	0.74 (0.53, 1.03)	0.74† (0.60, 0.94)
Upper East	0.64† (0.49, 0.84)	0.60‡ (0.50, 0.72)	0.80 (0.57, 1.13)	0.74* (0.59, 0.94)
Upper West	0.57‡ (0.42, 0.77)	0.71‡ (0.59, 0.86)	0.75 (0.52, 1.08)	0.96 (0.76, 1.21)
**Ethnicity:**				
Akan	Ref.	Ref.	Ref.	Ref.
Ewe	1.01 (0.86, 1.20)	1.01 (0.89, 1.14)	0.96 (0.77, 1.22)	1.03 (0.87, 1.22)
Ga Adangbe	0.93 (0.74, 1.17)	1.08 (0.92, 1.27)	0.78 (0.59, 1.02)	0.93 (0.77, 1.12)
Mole-Dagbani	0.59‡ (0.51, 0.69)	0.68‡ (0.61, 0.75)	0.86 (0.68, 1.08)	0.90 (0.77, 1.06)
Others	0.62‡ (0.52, 0.74)	0.69‡ (0.61, 0.77)	0.90 (0.72, 1.11)	0.96 (0.83, 1.11)

JNC – Joint National Committee, ACC/AHA – American College of Cardiology/American Heart Association; WD/DV/S – Widowed/Divorced/No Longer Living Together & Separated; HH/DD/S – Household and Domestic/Services/Skilled Manual/Unskilled manual; COR – Crude Odds Ratio; aOR – adjusted odds ratio; CI – confidence interval

*P < 0.05.

†*P* < 0.01.

‡*P* < 0.001.

Further, other risk factors considered under the ACC/AHA guideline were; occupation- agriculture (aOR = 0.81; 95% CI 0.68-0.96), Central (aOR = 0.82; 95% 0.69-0.98), Eastern (aOR = 0.80; 95% CI 0.66-0.96), Northern (aOR = 0.74; 95% 0.60-0.94) and Upper East (aOR = 0.74; 95% CI 0.59-0.94) regions recorded significant inverse relationships with hypertension. No significant effects were noted in ethnicity and the level of education (**Table 2****).**

### Differences in characteristics between males and females

The possible existence of difference in characteristics of the various risk factors across sex was investigated using χ^2^ test. The result showed that proportions of males across age categories (*P* < 0.001), level of education (*P* < 0.001), marital status (*P* < 0.001), place of residence

(*P* = 0.013) and occupation (*P* < 0.001) were significantly different. However, an even distribution of males and females was observed across the wealth quintile categories (*P* = 0.084), region (*P* = 0.561) and ethnicity (*P* = 0.328) (**Table 3**).

**Table 3:** Difference in characteristics between males and females, n (%)*

Characteristics	Female (n = 8295)	Male (n = 3856)	Overall (n = 12 151)	*P-*value
**Age (in years):**				
18-24	2226 (26.8)	977 (25.3)	3203 (26.4)	<0.001
25-34	2907 (35.0)	1074 (27.9)	3981 (32.8)	
35-44	2292 (27.6)	914 (23.7)	3206 (26.4)	
45-54	870 (10.5)	660 (17.1)	1530 (12.6)	
55+	0 (0)	231 (6.0)	231 (1.9)	
**Education:**				
No formal education	2231 (26.9)	641 (16.6)	2872 (23.6)	<0.001
Primary	1431 (17.3)	532 (13.8)	1963 (16.2)	
Secondary	4119 (49.7)	2239 (58.1)	6358 (52.3)	
College or above	514 (6.2)	444 (11.5)	985 (7.9)	
**Wealth quintile:**				
Poorest	1988 (24.0)	968 (25.1)	2958 (24.3)	0.084
Poorer	1522 (18.3)	738 (19.1)	2260 (18.6)	
Middle	1698 (20.5)	709 (18.4)	2407 (19.8)	
Richer	1598 (19.3)	737 (19.1)	2335 (19.2)	
Richest	1489 (18.0)	704 (18.3)	2193 (18.0)	
**Marital status:**				
Never in union	1969 (23.7)	1334 (34.6)	3303 (27.2)	<0.001
Married	4231 (51.0)	1967 (51.0)	6198 (51.0)	
Living with partner	1200 (14.5)	335 (8.7)	1535 (12.6)	
WD/DV/S	895 (10.8)	220 (5.7)	1115 (9.2)	
**Place of residence:**				
Urban	4131 (49.8)	1827 (47.4)	5958 (49.0)	0.013
Rural	4164 (50.2)	2029 (52.6)	6193 (51.0)	
**Occupation:**				
Not working	1587 (19.2)	326 (8.5)	1913 (15.8)	<0.001
Prof/Tech M/Clerical	567 (6.8)	505 (13.1)	1072 (8.8)	
Sales	2897 (35.0)	298 (7.8)	3195 (26.4)	
Agriculture	2009 (24.3)	1559 (40.6)	3568 (29.4)	
HH/DD/S	1220 (14.7)	1153 (30.0)	2373 (19.6)	
**Region:**				
Western	909 (11.0)	449 (11.6)	1358 (11.2)	0.561
Central	834 (10.1)	349 (9.1)	1183 (9.7)	
Greater Accra	912 (11.0)	428 (11.1)	1340 (11.0)	
Volta	714 (8.6)	312 (8.1)	1026 (8.4)	
Eastern	817 (9.8)	390 (10.1)	1207 (9.9)	
Ashanti	918 (11.1)	401 (10.4)	1319 (10.9)	
Brong Ahafo	867 (10.5)	430 (11.2)	1297 (10.7)	
Northern	921 (11.1)	423 (11.0)	1344 (11.1)	
Upper East	776 (9.4)	376 (9.8)	1152 (9.5)	
Upper West	627 (7.6)	298 (7.7)	925 (7.6)	
**Ethnicity:**				
Akan	3444 (41.5)	1549 (40.2)	4993 (41.1)	0.328
Ewe	1007 (12.1)	465 (12.1)	1472 (12.1)	
Ga Adangbe	474 (5.7)	252 (6.5)	726 (6.0)	
Mole-Dagbani	1960 (23.6)	913 (23.7)	2873 (23.6)	
Others	1410 (17.0)	677 (17.6)	2087 (17.2)	

WD/DV/S – Widowed/Divorced/No Longer Living Together & Separated; HH/DD/S – Household and Domestic/Services/Skilled Manual/Unskilled manual.

*Column percentages were taken. Inference was done using Pearson χ^2^ test.

## DISCUSSION

This study noted that, the overall prevalence of hypertension were 11.48% and 30.43% for the JNC7 and 2017 ACC/AHA guidelines, respectively. Considering the risk factors with both guidelines, we did not only show significant associations between age groups, urban residents and richer individuals, but also demonstrated sex-related difference in the determinants of hypertension, specifically, males exhibited higher BP levels compared to females. With regards to the individual guidelines, respondents in the middle income and richest wealth quintile groups, those engaged in agriculture, the widowed/divorced/no longer living together and separated as well as adults from Central, Eastern, Northern and Upper East regions demonstrated positive associations with the ACC/AHA 2017, whereas only manual or clerical workers were more likely to get hypertension according to the JNC7 guideline.

The proportion of Ghanaian adults’ population (18 years and above) classified as hypertensives significantly increased from 11.48% (JNC7) to 30.43% (2017 ACC/AHA) guidelines. This finding indicates that this condition affects a sizeable proportion of Ghanaian adults. The result according to the 2017 ACC/AHA guideline is substantially higher than the 13.0% prevalence rate of hypertension found by an earlier study among adults in Ghana [21]. Similar increases in prevalence according to the new guideline have been observed in recent studies elsewhere. A comparable pattern was seen in a study of hypertension prevalence among adults in US and China.

For the US study, adults’ population between the ages of 45 and 75 years classified as hypertensives increased from 49.7% (JNC7) to 63.0% (2017 ACC/AHA) guidelines. Likewise among Chinese adults of same age range identified as hypertensives increased from 38.0% to 55% based on same guidelines [16]. Similar substantial increments in prevalence were again observed in Bangladesh and Nepal [17,18]. As a result, this latest guideline brings to the fore the need to provide more health care infrastructure and logistics to manage the increased numbers of clients. In addition, new health screening strategies could be used to pick up potential clients early for prompt management.

Ageing is an independent predictor of hypertension. The result of our study is consistent with other Ghanaian and international studies [9,17-19,21-30]. The advanced age category of 55-64 years showed the highest likelihood of hypertension according to both guidelines. Thus, our result further confirms that ageing has a positive relationship with BP, and is a known risk factor of hypertension among the study population. The reason could be physiological ie, increasing age comes with several structural changes in arterial walls including increased responsiveness to sympathetic nervous system stimuli and increased cellular oxidative stress leading to arterial and arteriolar stiffness [30]. With old age as a predisposing factor of hypertension, the elderly is at a substantial risk for developing hypertension and other CVDs with consequent increased in disease burden on the health care system. Therefore, timely and regular interventions such as awareness creation to control and prevent hypertension with special focus on older adults should be emphasized.

Although, some previous studies have suggested that females were more likely to get BP compared to men [17,21,27,29], our study revealed that men were more likely to develop hypertension based on the cut-off points of both guidelines. This finding strengthens the notion that sex is a significant risk factor of BP. Other studies with the JNC7 and WHO algorithms’ have established similar findings in other parts of the world [23,24,26,28,30,31]. Though, both men and women develop hypertension, gender differences in the prevalence and severity of hypertension have been well established – where men have a higher prevalence of hypertension compared with women of the same age until the sixth decade of life. Again, the prevalence of hypertension for females in this study was lower than males at the younger ages (below 45 years), but it exceeded men of older ages. The above observation corroborates an earlier finding that estrogen hormone plays a protective role in women until menopause [32,33]. Both experimental and clinical studies have revealed that estrogen exerts different cardiovascular effects, such as sympatho-inhibition, vasorelaxation and subsequently decreasing aortic stiffness through actions on the endothelium and smooth muscle cells [34], thus, all these physiological activities act as protective factors against hypertension. Not only do the differences between sex hormones in both sexes contribute to this sexual dimorphism in BP, but the sex chromosomes also play a key role [35]. This suggests that the sex differences among adults may be partly due to biological differences, nevertheless, further studies are needed to investigate psychosocial factors that may contribute to this disparity. Most of the participants 52.3% had attained secondary school education, additionally, more males than females had secondary education and above. Although, there was a significant difference in educational level between males and females, there were no significant relationships between education and hypertension per both guidelines, which is similar to a finding by an earlier study in Ghana [21]. The crude odds ratio of subjects with college or above education seen in this study could be as a result of factors such as staying in urban areas.

Many educated persons in Ghana live in big towns and cities due to factors including job availability. On the other hand, these results are unlike a finding in Bangladesh with both cut-offs where hypertension showed significant relationship with higher education [17]. Whereas other studies found low level of education to be associated with hypertension [23,27,29,30,36]. The relationship between health and level of education could be complex, it has can be assumed that, the more educated a person is, the more knowledge he or she may have on matters of health. The truthfulness of this assumption did not reflect or otherwise in our study.

Several studies have reported an independent association between urban residence and hypertension [17,19,21,22,24,25,28,37]. In the present study, similar to the JNC7, urban residence had significant relationship with hypertension as per the 2017 ACC/AHA guideline. This could be due to the high psychological stress urban residents are subjected to due to factors such as heavy life demands, and traffic congestions experienced in cities [21], moreover, changes in lifestyle as a results of urbanization could be a factor, meanwhile, these challenges are expected to increase with continues urbanization [4].

The effects of household wealth status on hypertension have been confirmed in this study according to both guidelines. The risk of hypertension increases with increasing affluence according to 2017 ACC/AHA guideline. A previous study in Ghana revealed that an individual’s socioeconomic status may affect his/her behaviors and life choices, which could render him/her prone to getting hypertension. Similar observations have been reported elsewhere [19,21,31]. Further, hypertension was found to be more common among those who were widowed/divorced/no longer living together & separated (WD/DV/S) according to only the 2017 ACC/AHA guidelines.

The higher likelihood of hypertension among the WD/DV/S is similar to findings from previous studies [19,21,23,38] even though the reasons for this results is quite unclear, it could probably be as a results of inadequate income and inaccessibility to health care services due to low social support systems. Adults living in the Central, Eastern, Northern and Upper East regions had lower risk of hypertension, this is similar to an earlier finding [21]. The reasons for this finding is not clear, hence explorative studies are warranted to assess the reasons for the less likelihood of getting hypertension among inhabitants in these regions. Professional/technical, manual and clerical workers were independently associated with hypertension according to the JNC7 guidelines. This is consistent with another study which showed that higher efforts at work could be related to hypertension [39]. Therefore, workplace interventions that decrease exhaustion and stress should the implemented to reduce employees’ chances to get BP.

### Strengths and limitations

Our study used a nationally representative sample from all the administrative regions covering all the rural and urban areas of Ghana. This geographically large and diverse coverage makes the survey generalizable for Ghana. This survey used validated research instruments that further increased the authenticity of our results. The study had a high response rate. To our knowledge, this is the first survey to estimate hypertension prevalence among Ghanaian adults with the GDHS 2014 data according to the JNC7 cut off points and compared it with the risk factors as per the ACC/AHA 2017 guidelines.

The limitations of this study also warrant discussion. The GDHS was a cross-sectional study thus, causal pathways underlying the reported associations cannot be ascertained, and again, this technique might have resulted in some misclassification biases [17]. The skill level of some survey staff could also lead to misclassification bias [19]. Some known risk factors for hypertension such as stress, obesity, diabetes, physical activity, dietary habits, or dyslipidemia could not be investigated due to data set limitations.

## CONCLUSIONS

Early identification and treatment of people with hypertension is vital. Our study establishes more firmly the importance of identified risk factors of BP from the JNC7 algorithms utilizing the new 2017 ACC/AHA guideline definition. Overall, advancing age, urban residence, people with higher socioeconomic status, male sex, widowed/divorced/no longer living together & separated, agricultural, professional/ technical/manual and clerical workers need special interventions to manage the disease. We recommend similar studies in the future to follow up and describe trends in the risk factors; particularly, modifiable factors which are most likely be on the increase. We believe that these results would assist clinical care by determining the associations of these factors with hypertension in Ghana. Research on effectiveness on interventions are needed, and strategies to minimize the burden of hypertension risk factors should be replicated in Ghana if they have proven to be effective in similar settings. Our results shows the need for health policy makers to review the current hypertension guideline use in the country for better planning and implementation.

## Additional material

Online Supplementary Document
