# Supplementary material for: Increased prevalence of hypertension in Ghana: New 2017 American College of Cardiology/American Hypertension Association hypertension guidelines application
Source: J Glob Health. 2020 Sep 23;10(2):020408. doi: 10.7189/jogh.10.020408 (PMC7688293; doi:10.7189/jogh.10.020408)
Supplement: Online Supplementary Document [file jogh-10-020408-s001.pdf]

Table S1: Variables used in the study.

Variables	Description
Hypertension	JNC7: The systolic blood pressure (SBP) greater or equal to 140 mm Hg or diastolic blood pressure (DBP) greater or equal to 90 mm Hg or the respondent was taking any prescribed drugs to control blood pressure level<br>2017 ACC/AHA: The SBP greater or equal 130 mm Hg or DBP greater or equal 80 mm hg or the respondent was taking any prescribed drugs to control blood pressure
Age	Age of a respondent in years<br>Five Categories: 18-24, 25-34, 35-44, 45-54, 55-64
Sex	Sex of a respondent<br>Binary: Male and Female
Educational Level	Educational level of a respondent<br>Four levels: No formal education, primary, secondary, college or above
Marital Status	Marital status of a respondent<br>Four categories: Never in union, married, living together, Widowed/ divorced/ no longer living together/separated
Occupation	Occupation of a respondent<br>Five categories: Not working, professional or technical manager, clerical, sales, agriculture, and household & domestic/service/skilled manual/unskilled manual worker
Household Wealth Quintile	Household materials composite index obtained by principal component analysis.<br>Five strata: Poorest, poorer, middle, richer, and richest.
Place of residence	Rural or urban place of residence<br>Binary: Rural and urban.
Region	Region at which the individual is located<br>Ten categories: Western, Central, Greater Accra, Volta, Eastern, Ashanti, Brong Ahafo, Northern, Upper East, and Upper West.
Ethnicity	Ethnic group of a respondent<br>Five categories: Akan, Ewe, Ga-Adange, Mole-Dagbani, and others (Guan, Grusi, Gurma, Mande, and others)

ACC/AHA, American College of Cardiology/American Heart Association; JNC, Joint National Committee. Summary: This table describes the study variables investigated to examine determinants of hypertension as per two guidelines.
